# Herring-Based Diets Provide Robust Support for *Anopheles gambiae* Development and Colony Maintenance

**DOI:** 10.3390/insects17010101

**Published:** 2026-01-16

**Authors:** Samuel S. Akporh, Ibrahim K. Gyimah, Aaron A. Lartey, Samuel O. Darkwah, Godwin K. Amlalo, Sampson Gbagba, Ali Bin Idrees Alhassan, Godwin Hamenu, Dominic Acquah-Baidoo, Joannitta Joannides, Gladys N. Doughan, Godwin A. Koffa, Enyonam A. Akpakli, Akua O. Y. Danquah, Samuel K. Dadzie, Duncan K. Athinya, Rinki Deb, Rebecca Pwalia, Jewelna Akorli

**Affiliations:** 1 Vestergaard-NMIMR Vector Labs, Noguchi Memorial Institute for Medical Research, University of Ghana, Accra P.O. Box LG 581, Ghanadacquah-baidoo@noguchi.ug.edu.gh (D.A.-B.);; 2 Department of Parasitology, Noguchi Memorial Institute for Medical Research, University of Ghana, Accra P.O. Box LG 581, Ghana; 3 Vestergaard Frandsen (EA) Limited, 14 Riverside, Belgravia Block, 6th Floor, Nairobi P.O. Box 66889-00800, Kenya; 4 Vestergaard Sàrl, Place St. François 1, 1003 Lausanne, Switzerland

**Keywords:** *Anopheles gambiae*, larval diets, larval development, fecundity, population growth rate, insectary

## Abstract

Laboratory maintenance of mosquitoes is important for studying vector biology and transmission of diseases, and for testing vector control tools. Standard operating procedures require feeding larvae with commercial fish meal. However, for many insectaries in sub-Saharan Africa, the commonly used feeds are imported and accompanied by procurement challenges. Changing the larval feed abruptly without allowing the larvae to adapt to new brands of feed also leads to a decrease in mosquito colonies in the laboratory. We investigated locally acquired beans, maize, and dried herrings as alternate feeds for mosquito larvae reared under laboratory conditions. Four replicates for each treatment were prepared, each containing 100 first instar larvae of *Anopheles gambiae* Tiassalé mosquitoes. The larvae were introduced into 500 mL of dechlorinated tap water and maintained under standard environmental insectary conditions. The larvae were provided with 40 mg of the designated powdered feed—beans, maize, and herring fish—in single and combined treatments. Tetra^®^ goldfish meal was included as a control. The larval mortality, developmental time, and number of pupae were recorded to evaluate the effectiveness of the feeds. Adult mosquitoes were weighed and measured to assess fitness, and females from each treatment were blood-fed and allowed to lay eggs to evaluate fertility. Larval survival differed significantly across diets (Kruskal–Wallis, *p* = 0.01), with maize-fed larvae showing the highest mortality (41.3%) and those with herring-based diets the lowest. Pupation and adult emergence were poorest in the maize and maize–bean groups, while the maize–herring combination achieved the highest emergence (92.6%, *p* = 0.03). Although overall differences were detected among the groups, conservative pairwise tests did not pinpoint specific group contrasts, but effect size estimates suggested biologically meaningful patterns. Generally, adult body weight and wing length did not differ by treatment except in maize-fed males (β = 0.371 mm, *p* = 0.022). Herring fish-based diets consistently supported larval survival, timely development, and robust fecundity, whereas maize-based diets were nutritionally inadequate. These findings highlight herring fish-based diets as a sustainable and cost-effective alternative to commercial feeds for maintaining *Anopheles* mosquito colonies, with potential to strengthen vector research capacity in resource-limited laboratories.

## Simple Summary

The maintenance of mosquitoes in the laboratory is important for vector biology research. Obtaining healthy adult mosquitoes starts with ensuring that the larvae are fed well. The established standard protocols, however, suggest the use of feeds that are usually commercial, presenting procurement challenges for laboratories, particularly in low-resource settings. Therefore, this study assessed the use of local produce, including maize, beans, and dried herrings, as feed for mosquito larvae. We tested how well these feeds supported the development of the larvae, and tested their effect on adult size, weight, and the ability of the female adult to reproduce the next generation of offspring. Our results showed that herrings performed best among all feeds tested and were comparable to the commercial feed. Although beans often resulted in lower estimates, the negative effect could be reduced with the addition of herrings. We conclude that herrings alone or herring-based feeds that are readily available, cheap, and sustainable can be used in laboratory maintenance of *Anopheles* mosquitoes. Further studies can improve on this by standardizing proportions of herring and maize and/or bean feed combinations for optimal mosquito maintenance for research.

## 1. Introduction

The WHO prequalification process for evaluating and accepting the use of new vector control tools requires that they be tested against mosquitoes in a controlled environment [1]. Lab-reared mosquitoes play an important role in giving firsthand scientific evidence of the effect of a control tool in reducing disease transmission, providing a strong rationale for the wide-scale implementation of interventions. Laboratory rearing of mosquitoes also helps researchers to study and understand the behaviour of mosquitoes and how they react to the tools being tested [2].

During colonization and mass production of *Anopheles* mosquitoes, several factors can influence the survival of the mosquito colony. These include larval density, diet, temperature, and environmental microbiota [3]. Larval nutrition is an important consideration in raising mosquitoes for research purposes. The quantity and quality of larval feed are important in the development, survival, and adult emergence of laboratory-raised mosquitoes [4]. The lack of adequate nutrition during the larval development stage of mosquitoes can result in delayed or failed development, or production of adults with adverse phenotypes [5]. For successful development of larvae into adults, mosquito larvae require sugars, nucleotides, polyunsaturated fatty acids, sterols, vitamins, and fourteen essential amino acids [6]. Larval diets should provide a wide range of nutrients to accommodate the nutritional requirements for larval development.

Commercial feeds commonly recommended for raising mosquito larvae in the lab include Tetramin^®^ fish food, which is the most widely used option [7,8,9,10,11,12]. Other diets that have been used include dog, rodent, and cat food or a combination of diets with ingredients from cereals and legumes mixed with liver [13,14,15]. For insectaries outside of the Northern Hemisphere, these commercial products used as larval feeds are imported, making them expensive and logistically challenging. Introducing changes to the brand of feed can slow down production, as lab-reared mosquitoes require some time to adapt to the new feed. This study, therefore, aims to evaluate the potential use of locally sourced feed options for larval rearing in insectaries that face challenges in obtaining readily available commercial feeds. We investigated the development of lab-reared *Anopheles gambiae* mosquitoes after feeding larvae with maize, beans, and dried herrings to identify cost-effective and readily available feeds that will provide no loss in productivity compared to commercial feeds.

## 2. Materials and Methods

### 2.1. Study Design

The feeds tested in this comparative study were maize (*Zea mays*), cowpea/black-eyed beans (*Phaseolus vulgaris*), and herrings (*Clupea harengus*): maize (M), beans (B), dried herrings (H), and combinations of dried herrings and beans (HB), maize and dried herrings (MH), maize and beans (MB), and beans, maize, and herrings (BMH). Tetra^®^ goldfish flakes (Tetra GmbH, Melle, Germany) were used as the control feed. All combinations were constituted in equal amounts. The parameters used to assess performance of the feeds were larval and pupal survival, adult emergence, weight and wing measurements of adult mosquitoes, blood-feeding success, and fecundity of female adults.

### 2.2. Feed Preparations

Maize, beans, and dried herring were obtained from the local market and finely blended into powder free of any visible lumps. The powdered maize, beans, and herring were further sifted using a 20-mesh sieve with an opening size of 850 µm to ensure that the feed was fine in texture to enable easy dissolution in the larval breeding water. For the combined feed treatments, equal proportions of each feed ingredient were thoroughly blended to ensure a uniform mixture. All feeds were preserved in an air-tight plastic container and stored in the fridge at 4 °C until use.

### 2.3. Assessment of Feed Performance

The *Anopheles gambiae s.l.* Tiassalé strains were used in this study. These strains originated from Cote d’Ivoire and have been maintained in the Vestergaard-Noguchi Vector Labs in Accra, Ghana (VNVL), since 2010 [16]. The lab colonies were raised in dechlorinated water and maintained on Tetramin^®^ fish flakes (Tetra GmbH, Melle, Germany) until they were separated into treatments for this study.

Eggs from the lab-maintained *Anopheles gambiae* Tiassalé strain were hatched in dechlorinated tap water with yeast. Four replicates for each treatment were prepared, each containing one hundred (100) first instar larvae of *Anopheles gambiae* Tiassalé mosquitoes. The larvae were introduced into 500 mL of dechlorinated tap water and maintained under standard insectary conditions of 27 ± 2 °C and a relative humidity of 75 ± 10%. The larvae were fed daily with 40 mg of their designated feed treatments. Controls were fed similarly with Tetra^®^ goldfish flakes. Larval bowls were checked daily to record larval mortality and the number of pupae. The pupae picked from each bowl were placed in their respective cages and monitored for adult emergence.

Next, 25 females aged 3–5 days were aspirated from each cage into smaller cages and sugar-starved for an hour. Each cage was fed with defibrinated sheep blood using a Hemotek membrane feeder for two consecutive days to assess the success of blood feeding of female adults from each feed treatment. To collect eggs, oviposition trays were provided 48 h after the first blood meal was offered. Eggs laid were counted and recorded.

To assess mosquito weight across treatments, 100 unfed females and 100 males were sampled from each treatment, with an equal number of mosquitoes drawn from all four replicates for each treatment. The mosquitoes were sedated using chloroform and weighed with a Mettler balance (Mettler Toledo, Columbus, OH, USA). After weighing, 50 female and 50 male mosquitoes were sampled from each treatment for wing measurement. The wings of the mosquitoes were removed and fixed to a microscope slide, and a photo was taken. The length of the wings from the distal edge of the alula to the end of the radial vein (excluding fringe scales) was estimated using Wingy software (version 14/08/22), developed by the Liverpool School of Tropical Medicine.

### 2.4. Data Analyses

The data were not normally distributed after they were assessed using the Shapiro–Wilk test; therefore, subsequent analyses were performed using non-parametric tests. Overall group comparisons were performed with the Kruskal–Wallis test and Dunn’s post hoc test with Benjamini–Hochberg corrections. Where overall group significance was observed but Dunn’s test failed to show pairwise differences, a rank-biserial correlation analysis was conducted to investigate potential biological relevance. Here, the threshold for significant effect size was set at >0.5 and <−0.5. The Wilcoxon rank sum test was used to compare weight and wing lengths between male and female adult mosquitoes.

Regression models were fit to determine the relationship between variables. Linear regression models were used unless assumptions of the standard linear regressions were not met. A quasi-binomial generalized linear model was fit to assess the effect of treatment on larval mortality because the dependent variable was calculated as the successes and failures. In instances where response variables were proportions or percentages—for example, adult emergence (the proportion of larvae that became adults)—a beta regression model was used. All analyses were performed and, visualization plots were created using custom scripts in R-statistical software (version 4.5.1).

## 3. Results

### 3.1. Effect of Feed on Mosquito Development

**Figure 1:**
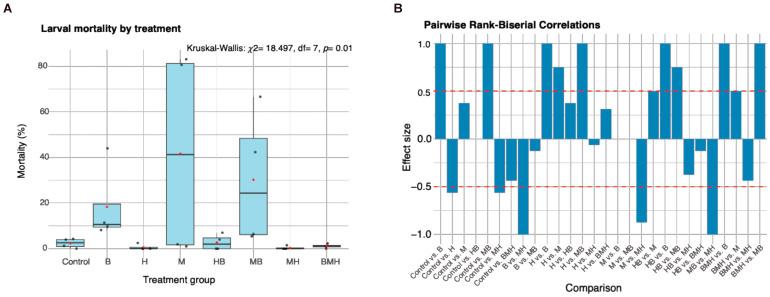
The effect of feed treatments on larval mortality. Box plot (**A**) illustrating the median (black horizontal line in each box) and distribution of larval mortality percentages for each treatment group. The black dots represent mean larval mortality for each replicate and red dots represent the treatment group mean. (**B**) displays the pairwise rank-biserial correlations showing the effect size of each treatment comparison. A positive value indicates higher mortality in the group listed second compared to the group listed first, and a negative value indicates lower mortality in the second group. The dashed red lines represent the thresholds for a medium effect size (r = ±0.5).

Median larval mortality ranged between 0% in herring (H)-only and maize–herring (MH) combination treatments, and 41.3% in those fed on maize (M) only. Larval mortality differed significantly among all groups (Kruskal–Wallis: *p*= 0.01) (Figure 1A), but results from Dunn’s post hoc test for pairwise comparison showed no adjusted *p*-value was < 0.05 (Table S1). This indicates that no single pair of groups was different enough to reach the threshold for statistical significance. Rank-biserial correlation was therefore used to investigate this further, to measure the effect size and quantify the strength of the relationship between treatment and larval mortality. Values range from −1 to 1, where a value closer to the extremes indicates a stronger effect, and a value closer to 0 indicates a weak effect. According to this analysis, beans (B) and maize–beans (MB) were the only treatments with a stronger effect on mortality (effect size = 1) compared to the control (Figure 1B). Generally, feeds that did not contain herrings correlated with increased larval mortality compared to feeds with herrings, with effect sizes ranging from 0.75 (e.g., H vs. M) to 1 (e.g., H vs. B). Herrings (H) and maize–herrings (MH) showed lower effect mortality (effect size= −0.56) compared to the control, suggesting that herring feeds performed marginally better than the control (Figure 1B).

**Figure 2:**
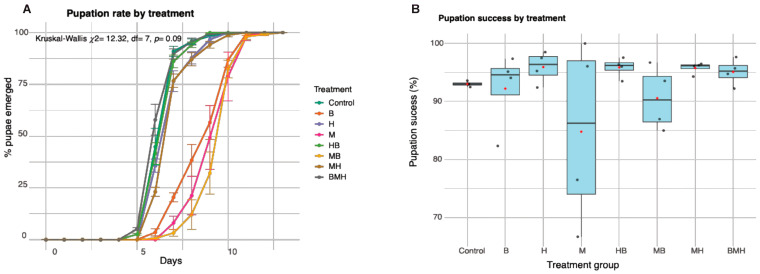
Pupation rates and success by treatment groups. A line plot (**A**) shows the percentage of pupae that emerged over time (in days) for each treatment group. Error bars represent standard deviation. The box plot (**B**) illustrates the median (black horizontal line in the box) and distribution of overall pupation success percentages for each treatment group. The black dots represent mean larval mortality for each replicate and red dots represent the treatment group mean.

Pupation rates did not differ between treatments (Kruskal–Wallis *p*= 0.09), although larvae fed on beans (B), maize (M), and their combination (MB) appear to lag in their pupation by 1–2 days compared to the control (Figure 2A). The treatments (M and MB) that caused high larval mortality also resulted in low pupal success rates (Pearson: *r*= −0.89, *p* < 0.0001) (Figure 2B).

**Figure 3:**
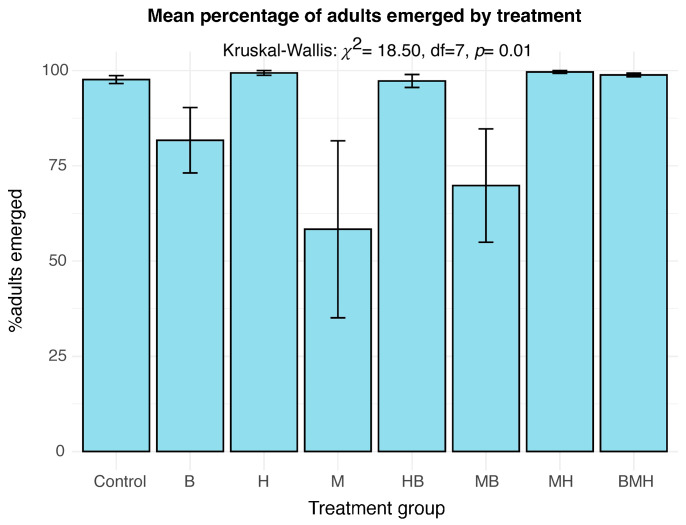
The success of adult emergence by treatment groups. Bar plots show the mean emergence success (%) for each treatment group. The error bars represent the standard error of the mean.

The number of adults emerging was calculated as a percentage of the total larvae introduced to the feed to determine the effect of feed on adult outcome (emergence success). The mean adult emergence success ranged between 58.3% (M) and 99.6% (MH) (Figure 3). While there was overall significance between treatment groups (*p* = 0.010), adjusting for multiple testing in pairwise comparisons using Benjamini–Hochberg corrections showed no significant difference between any treatment pair (*p*.adj > 0.05) (Table S2), like earlier observations in this study. The beta regression generalized linear model results, however, demonstrated that herrings (H) and maize–herrings (MH) were the only feeds that positively influenced the final adult emergence success relative to the control (Table 1). All other treatments produced a reducing effect on adult emergence compared to the control, the strongest effect being maize–beans (MB) (β = −0.775). BMH treatment had a similar effect to the control (*p* = 0.71). In addition, the proportion of adults emerging was positively dependent on the success of the larval stage (i.e., pupae) (β = 0.0667, *p* < 0.0001) and not the number of larvae exposed to the treatment (Table 1).

### 3.2. Effect of Treatment on Population Growth

**Figure 4:**
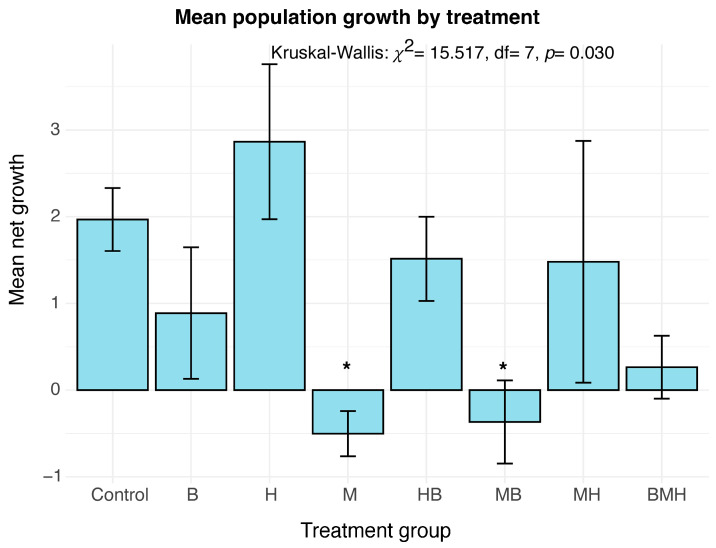
Net population expansion by larval diet. Bar plots show the net growth calculated by subtracting the number of founding larvae from the number of F1 adults and dividing by the number of founding larvae. The error bars represent the standard error of the mean. Treatments with significant (*p* < 0.05) mean net growth compared to Control are shown with asterisks (*).

The net population growth was calculated as the ratio of the change in population (F1 adults minus initial larvae) to the initial number of larvae. To investigate the effect of treatment on population growth, a linear model (lm) analysis and a non-parametric Kruskal–Wallis test were performed. The linear model showed a significant overall effect of treatment (F(7,24) = 2.662, *p* = 0.034). Post hoc analysis of the linear model revealed that M (estimate = −2.471, *p* = 0.023) and MB (estimate = −2.336, *p* = 0.030) significantly reduced population growth compared to the control (Figure 4). Like previous results, there was a difference in net growth between groups (Kruskal–Wallis: *p* = 0.030), but Dunn’s post hoc test with correction did not show any treatment that was significantly different from the control (Table S3). The discrepancy between the two models likely stems from the fact that the linear model relies on assumptions of normality and equal variance, while the Kruskal–Wallis test is more conservative and relies on ranks. Given the significance found in the linear model and the clear negative trend in the M and MB groups (Figure 4), the linear model provides a strong indication of a real effect.

### 3.3. Assessment of Adult Fitness

**Figure 5:**
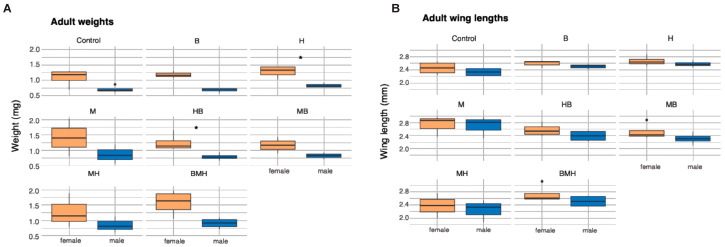
Weight and wing length comparison between male and female adults for each feed treatment. Box plots illustrate the distribution of adult weights (mg) (**A**) for male and female mosquitoes across different treatment groups. The distribution of adult wing lengths (mm) for male and female mosquitoes across different treatment groups is shown in box plots (**B**). An asterisk (*) above a box plot indicates a significant difference between the sexes in that treatment group based on a Wilcoxon rank sum test.

Weight, wing length, and fecundity were used as indicators for assessing the fitness of the adult mosquitoes that emerged from each treatment. The weights of males and females were similar across treatments (*p* > 0.05). Males weighed on average 0.81 mg (range = 0.56–1.20) while females weighed 1.28 mg (range = 0.70–2.03) (Wilcoxon: W = 656.5, *p* < 0.0001). The difference between male and female weight was more distinct (Wilcoxon: W = 16, *p* = 0.03) within the herring (H) and herring–bean (HB) treatments (Figure 5A). Adult male wings ranged between 1.88 and 2.97 mm (mean = 2.44 mm) while female wing lengths were between 2.00 and 3.12 mm (mean = 2.58 mm) (Wilcoxon: W = 574, *p* = 0.02). Contrary to weight, H and HB treatments did not show differences in male and female wing length.

Overall, treatment did not influence male wing length (lm: F(7,21) = 2.009, *p* = 0.10), but maize (M) treatment resulted in a 0.371 mm increase in male wing length compared to the control (β = 0.371, SE = 0.150, t (21) = 2.478, *p* = 0.022). On the other hand, females showed no differences in their wing lengths across treatments (F(7,21) = 0.964, *p* = 0.48). Females’ wings were longer than males’ in MB and BMH treatments (Figure 5B).

**Figure 6:**
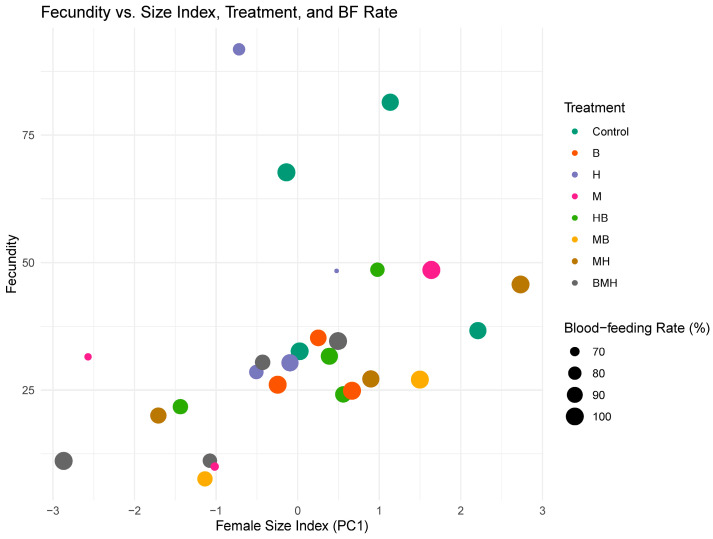
Bubble plot of the relationship between the fecundity, size index, treatment, and blood-feeding rate of female adults. The female size index was obtained from first principal component (PC1) analyses of weight and wing lengths. Each point represents a replicate, and the colour of each point indicates its treatment group. The size of the point corresponds to the blood-feeding rate, which was determined as the proportion of emerged females that blood-fed.

Using principal components, the weights and wing lengths of females were transformed into a ‘size index’ that is a linear combination of both variables. A negative binomial regression model was then fit to test the influence of size, blood-feeding rate (proportion of blood-fed females), and treatment on fecundity (average eggs laid per blood-fed female). The model explained 54.25% (pseudo-R^2^ = null deviance—residual deviance) of the observed variance in fecundity. Size was a positive predictor of fecundity (β = 1.215, *p* = 0.0048), while all test treatments, except herrings (*p* = 0.89), showed a negative effect (Table S4). Some treatments (control, H, and HB) appeared to be spread across a wider range of sizes and fecundities while others were more clustered (Figure 6). Higher blood-feeding rates were also observed to associate with some of the highest fecundity values. Among the treatment groups, only the MB (maize–beans) treatment showed a significant negative effect on fecundity compared to the control (β = −34.76, SE = 16.07, t(17) = −2.163, *p* = 0.045).

## 4. Discussion

This study assessed the impact of selected readily available local produce as larval diets on key life history traits of laboratory-reared *Anopheles* mosquitoes. Our results demonstrated that the feeds supported mosquito development, with varied effects on important fitness metrics, particularly fecundity, adult weight, and population expansion. Overall, herring and maize–herring diets were most comparable to the commonly used commercial feed (control), sometimes performing slightly better than the latter based on effect size analyses, which demonstrated significant biological relevance. These feeds can be used as alternatives in the maintenance of mosquito populations in the insectary following further studies on how they meet larval nutritional needs, and product standardization.

First, larval mortality was comparatively more pronounced with maize feeds, while the herring and the maize–herring diets exhibited a near-zero median mortality. During the experiment, the breeding water for the maize-fed group turned cloudy over time, which possibly reduced the oxygen availability for the larvae by interfering with gas exchange on the water surface, making the water unfavourable for larval survival [17,18]. Larval mortality was highly variable in the maize-alone group, but the addition of herrings (MH) improved larval survival comparable to the herring-alone treatment. Protein plays a critical role in mosquito larval growth, but excessive protein intake has been reported to slow or even stunt larval development in some cases [19]. Herrings are highly rich in proteins and provide a wide array of other nutrients, including omega-3 fatty acids, vitamins, and selenium, that may be critical for mosquito development and buffer against the negative effects of excessive protein [20]. The balanced nutrient profile of the herrings, particularly their fatty acids, supports membrane synthesis and energy metabolism, which promotes larval survival and development [21]. On the other hand, plant-based proteins could have flavonoids and alkanoids that inhibit important insect growth enzymes [13,22,23]. We observed that bean-only diets resulted in significantly high larval mortality compared to other bean meal combinations and the control group, confirming the larvicidal effects of this legume [24].

Larvae reared in nutrient-deficient environments attempt to prolong development to accumulate sufficient reserves for metamorphosis, resulting in smaller or less fit adults [25,26]. We, however, observed no difference in the pupation rates across treatments. Larvae fed on maize and the maize–bean combination exhibited the lowest and most variable pupation success, although this was not observed to be significant. This was not surprising since pupae do not feed, and survival was not dependent on nutrition at this stage. Adult emergence was dependent on the success of the larval rather than the pupal stage, highlighting the importance of early-stage nutrition in determining the availability of adult mosquitoes. Nutrient-rich larval diets, particularly those rich in animal protein, have been shown to produce higher emergence success and fitter adults compared to plant-based diets alone [13,19]. Our results, however, showed that the effect of the tested diets on weight and wing lengths was not sufficient to observe differences from mosquitoes reared on the commercial feed. Notably, the adult females were of similar weights and wing lengths to adult males across most treatments, including the control. Only herring (H) and herring–bean (HB) diets produced significantly heavier females than males. Herring appears to sufficiently support sexual dimorphism in adult mosquitoes and could be playing a key role in female accumulation of reserves for egg production [27]. Previous studies have shown that fish-pellet diets support mosquito larval development in laboratory settings and promote faster growth, reflecting their high nutritional quality in insectary rearing protocols [24], a trend that was also observed in the present study.

We combined weight and wing lengths into a composite size index to allow better assessment of their effects on fecundity. A key finding, consistent with established mosquito biology, was the significant positive relationship between female composite size and fecundity [28,29]. This underscores the principle that larger females accumulate greater resource reserves necessary for vitellogenesis, and that nutritional quality during the larval stage determines the adult female’s capacity to produce eggs. This strong association between size and reproductive output suggests that any diet-induced reduction in adult size inevitably translates into lower potential for population expansion. The negative binomial generalized linear mixed model (GLMM), controlling for both size index and blood-feeding rate, revealed that several diets significantly reduced fecundity compared to the control group. The highest reduction in fecundity compared to the control was in the maize–bean (MB) treatment, which reduced predicted fecundity by ~32%. The herring diet performed similarly to the control, showing a wider range of fecundity with a higher-than-average body size (Figure 6). These findings confirm that poor larval nutrition may not always manifest as smaller size, but can nonetheless compromise reproductive output, consistent with previous reports where larval stress reduced ovarian development and egg production even in normal-sized females [30]. Our model also found that the blood-feeding rate was not a significant predictor of fecundity when size and larval diet were accounted for. Variations in the quality of the larval diets were more important than the rate of successful blood meals among the groups and the resulting female body size in determining overall egg batch size.

The detrimental effects observed on individual life history traits translated directly into population-level outcomes. Analysis by the linear model identified that maize (M) and maize–bean (MB) treatments significantly suppressed the population net rate by 125% and 118%, respectively, compared to the control group. The significantly lower rates in these groups suggest a failure to support exponential growth, likely due to a combination of reduced larval survival rates and fecundity.

We acknowledge that the lack of information on the history of the farm produce used in this study could potentially have affected the performance of the tested diets. This gap in information means that potential residues from fertilizers, insecticides, or other contaminants present in the feeds could not be accounted for. These factors need to be considered in subsequent studies as they may influence the survival of the larvae. Subsequent work could investigate how these local larval diets may influence the physiological traits linked to insecticide resistance, which is a key phenotype that is maintained in selected lab-bred mosquito strains. Since the larval fat body plays a central role in regulating hormones and nutrient metabolism, it may also affect the expression of detoxification enzymes critical for resistance mechanisms [24]. Further studies should (a) assess whether these alternative diets modulate enzyme activity related to insecticide susceptibility, (b) assess biochemical standardization of these diets, and (c) evaluate the long-term effects on key adult traits such as mating competitiveness, vector competence, and insecticide resistance over multiple generations. Ensuring that mosquitoes reared on alternative feeds remain biologically comparable to those raised on standard diets will be essential for both laboratory-based research and operational vector control programs.

A key limitation of this study was the failure of Dunn’s pairwise comparison tests to achieve statistical significance for individual comparisons following significant Kruskal–Wallis tests. While the latter test confirms dietary effects were indeed present, the lack of significant pairwise differences suggests that the dietary effect was broadly distributed across all treatments, preventing the isolation of a single, strongly differentiated pair. Hence, our ability to rank individual diets is limited by the statistical power required for conservative post hoc testing. A larger sample size within groups and/or more replicates could be used to draw out clear differences between treatments.

## 5. Conclusions

Findings from this study have shown the effectiveness of a less expensive alternative for feeding mosquitoes for mass rearing. Pure herrings proved best for mass rearing of *Anopheles gambiae* mosquitoes under laboratory conditions. Adoption of herring-based diets could significantly improve the sustainability of mosquito rearing programs in resource-limited laboratories, thereby strengthening the capacity to conduct bioassays and vector control research in malaria-endemic regions.

## Figures and Tables

**Table 1:** Beta regression analysis of adult emergence success. The table shows beta coefficients (β), 95% confidence intervals (CIs), and *p*-values from the beta regression model [betareg(emergence_proportion ~ tmt + pupae + total.larvae)]. The control group serves as the reference level for the treatment variables.

Characteristic	β-Estimate	95% CI	*p*-Value
B	−0.69	−1.1, −0.26	0.002
H	1.4	0.50, 2.2	0.002
M	−0.56	−1.1, −0.04	0.036
HB	−0.64	−1.0, −0.25	0.001
MB	−0.77	−1.1, −0.41	<0.001
MH	1.1	0.27, 1.8	0.008
BMH	−0.11	−0.70, 0.48	0.7
Pupae	0.07	0.06, 0.08	<0.001
Total larvae	−0.04	−0.05, −0.02	<0.001

## Data Availability

The original contributions presented in this study are included in the article/Supplementary Material. Further inquiries can be directed to the corresponding authors.

## Supplementary Materials

The following supporting information can be downloaded at: https://www.mdpi.com/article/10.3390/insects17010101/s1, Table S1: Results of Dunn’s pairwise comparison and rank-biserial correlation for larval mortality across tested diets. Table S2: Results of Dunn’s pairwise comparison for pupation across tested diets. Table S3: Results of Dunn’s pairwise comparison for net population growth across tested diets. Table S4: Model results for negative binomial generalized linear mixed model assessing influence of composite size index of adult female mosquitoes, diet, and blood-feeding rate on fecundity.

## Abbreviations

The following abbreviations are used in this manuscript:

B	beans
H	herrings
HB	herrings–beans
M	maize
MB	maize–beans
MH	maize–herrings
BMH	beans–maize–herrings
